# Novel water-soluble polyurethane nanomicelles for cancer chemotherapy: physicochemical characterization and cellular activities

**DOI:** 10.1186/1477-3155-10-2

**Published:** 2012-01-05

**Authors:** Ahmad Yari Khosroushahi, Hossein Naderi-Manesh, Hamid Yeganeh, Jaleh Barar, Yadollah Omidi

**Affiliations:** 1 Faculty of Bioscience, Tarbiat Modares University, Tehran, Iran; 2 Research Centre for Pharmaceutical Nanotechnology, Faculty of Pharmacy, Tabriz University of Medical Sciences, Tabriz, Iran; 3 Faculty of Polymer Science, Iran Polymer and Petrochemical Institute (IPPI), Tehran, Iran; 4 Ovarian Cancer Research Center, Perelman School of Medicine, University of Pennsylvania, Philadelphia, PA 19104, USA

**Keywords:** Bioactive biocompatible polymer, Cancer chemotherapy, Nanomicelle, Nanoparticle, Polyurethane, Paclitaxel

## Abstract

**Background:**

Efficient delivery of anticancer chemotherapies such as paclitaxel (PTX) can improve treatment strategy in a variety of tumors such as breast and ovarian cancers. Accordingly, researches on polymeric nanomicelles continue to find suitable delivery systems. However, due to biocompatibility concerns, a few micellar nanoformulations have exquisitely been translated into clinical uses. Here, we report the synthesis of novel water-soluble nanomicelles using bioactive polyurethane (PU) polymer and efficient delivery of PTX in the human breast cancer MCF-7 cells.

**Results:**

The amphiphilic polyurethane was prepared through formation of urethane bounds between hydroxyl groups in poly (tetramethylene ether) glycol (PTMEG) and dimethylol propionic acid with isocyanate groups in toluene diisocyanate (TDI). The free isocyanate groups were blocked with phenol, while the free carboxyl groups of dimethylol propionic acid were reacted with triethylamine to attain ionic centers in the polymer backbone. These hydrophobic PTMEG blocks displayed self-assembly forming polymeric nanomicelles in water. The PTX loaded PU nanomicelles showed suitable physical stability, negative zeta potential charge (-43) and high loading efficiency (80%) with low level of critical micelle concentration (CMC). In vitro drug release profile showed a faster rate of drug liberation at pH 5.4 as compared to that of pH 7.4, implying involvement of a pH-sensitive mechanism for drug release from the nanomicelles. The kinetic of release exquisitely obeyed the Higuchi model, confirming involvement of diffusion and somewhat erosion at pH 5.4. These nanomicelles significantly inhibited the growth and proliferation of the human breast cancer MCF-7 cells, leading them to apoptosis. The real time RT-PCR analysis confirmed the activation of apoptosis as result of liberation of cytochrome c in the cells treated with the PTX loaded PU nanomicelles. The comet assay analysis showed somewhat DNA fragmentation in the treated cells.

**Conclusions:**

Based upon these findings, we propose that the bioactive waterborne polyurethane nanomicelles can be used as an effective nanocarrier for delivery of anticancer chemotherapies such as paclitaxel.

## Background

Paclitaxel is an anticancer drug that has successfully been used against a variety of tumors such as ovarian and breast cancers [1]. It is a lipophilic agent with limited solubility in water (0.3 μg/ml) [2], thus the most available injectable formulation of paclitaxel (i.e., Taxol^®^) has been formulated using mixture of Cremophor EL and ethanol (1:1 v/v). However, such formulation was reported to induce serious undesired adverse reactions such as neurotoxicity and anaphylactic reactions due to high amount of Cremophor EL [3] that can also alter the pharmacokinetics of PTX [4].

So far, various formulations of PTX have been developed to enhance the aqueous solubility of PTX and to obtain suitable delivery system for controlled release and/or targeted delivery of PTX [5-9]. Of these, biodegradable and biocompatible hydrotropic polymeric nanocarriers (50-200 nm) with an efficient loading and solubilizing potential were shown to accumulate in tumor microenvironment through enhanced permeation and retention (EPR) effects [5,6,10]. Having exploited passive targeting strategy based on EPR effects, the major objectives of these studies were to achieve a better pharmacokinetic profile with maximum efficiency in cancer cells and minimum toxicity in normal cells/tissue.

For Cremophor EL-free micellar formulation of paclitaxel, various block copolymers have been previously exploited including: diblock copolymer methoxy poly(ethylene glycol)-block-poly (D, L-lactic acid) [11], methoxy poly(ethylene glycol)-block-poly(caprolactone) [12,13], chitosan-poly(epsilon-caprolactone)-poly(ethylene glycol) [9], chitosan-poly(epsilon-caprolactone)-poly(ethylene glycol) graft copolymers [9], hydrotropic oligomer-glycol chitosan [14], cyclic RGD conjugated poly (ethylene glycol)-co-poly (lactic acid) [15], a core-shell-type' polymeric micellar nanoparticle formulation (NK105) [16,17], poly (ethylene glycol)-poly(4-(2-vinylbenzyloxy-N-picolylnicotinamide) [18], poly(2-ethyl-2-oxazoline)-block-poly(epsilon-caprolactone) [19], poly (lactic acid)-poly (ethylene oxide)-Arg-Gly-Asp [20], and triblockpolylactic acid (PLA)- polyethylene glycol(PEG)-PLA [21]. For instance, polymeric micelle-entrapped PTX (Genexol-PM) has recently been approved for clinical use [22-24]. This micellar nanostructure is not aggregated or taken up by reticuloendothelial system (RES).

Having considered the biocompatibility of polyurethane polymers and their wide applications in biomedical consumption [25,26], in the current work, we synthesized a novel waterborne polyurethane micellar nanoformulation and studied its potential for delivery of PTX in the human breast cancer MCF-7 cells.

## Results

### Spectrophotometric characterization of PUD

**Figure 1:**
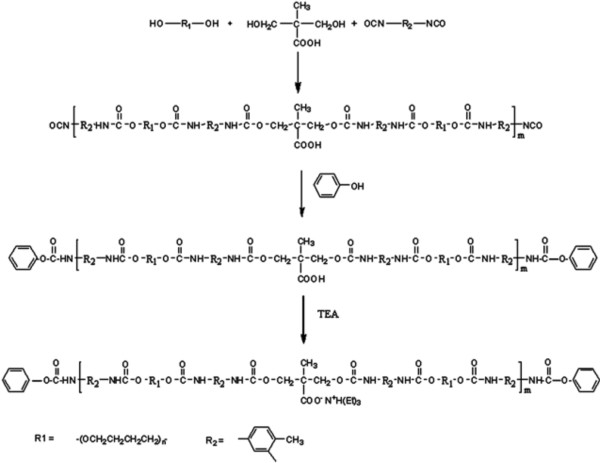
**Synthesis scheme of polyurethane dispersion**.

**Figure 2:**
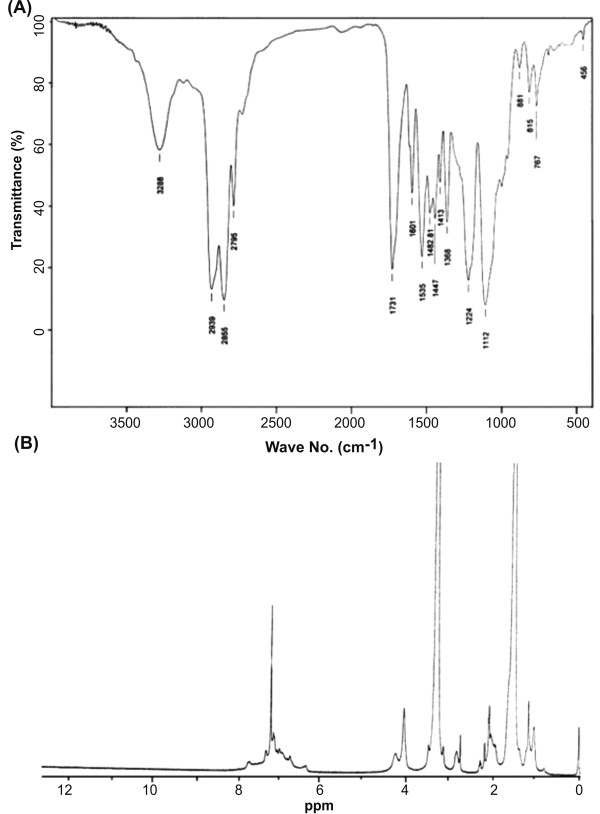
**FTIR (A) and ^1^HNMR (B) spectra of PUD**.

We used an optimized synthesis methodology for production of polyurethane dispersion (Figure 1). The structure of the prepared phenol blocked polyurethane dispersion (PBPUD) was confirmed by FTIR spectroscopy (Figure 2A). The absence of characteristic NCO absorption around 2270 cm^-1 ^indicates the absence of free NCO groups. These data suggest that the NCO groups were effectively blocked by phenol. Strong absorptions at 1731 cm^-1 ^(C = O stretching of urethane and carboxylic groups), 2850 and 2939 cm^-1 ^(CH_2 _stretching vibrations of PTMEG and TDI), 1112 cm^-1 ^(C-O-C stretching vibration of PTMEG), 3288 cm^-1 ^(N-H stretching), 1533 cm^-1 ^(N-H bending) and 1210-1240 cm^-1 ^(the stretching vibration of the C = O group combined with the N-H group) confirmed the PBPUD formation.

In ^1^HNMR spectrum of polyurethane, the internal methylene groups of PTMEG and methyl groups of triethylammonium moieties were detected at 1.59 ppm. The CH_3 _group of TDI was observed at 2.14 ppm. The groups attached to etheric oxygen atom and methylene groups of triethylammonium moieties appeared at 3.32-3.53 ppm. The peaks at 4.13 and 4.31 ppm represent methylene groups of PTMEG and DMPA moieties connected to urethane oxygen atom. Multiplet peak observed at 6.78-7.73 ppm was attributed to aromatic protons of TDI and phenol, as well as urethane NH groups (Figure 2B).

### Size, size distribution and zeta potential analysis

**Figure 3:**
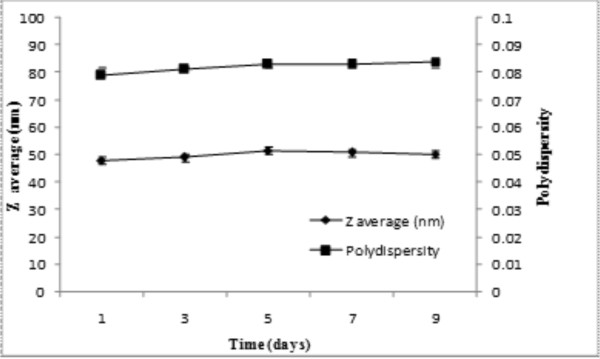
**Average diameter and polydispersity of PUD nanomicelles**. Data represent replicates at room temperature.

As shown in Table 1, the average hydrodynamic diameter of PTX loaded micelles and their polydispersity were respectively 50.3 ± 1.3 nm and 0.074 ± 0.006 nm, while they showed average zeta potential about -42.98 ± 1.53 mV. The pyrene partition study displayed a CMC value of 0.2 mg/L for PUD polymer in aqueous solutions (Table 1). These nanostructures retained their average size and polydispersity over 9-day incubation period at room temperature (Figure 3).

**Table 1:** Physicochemical characteristics of PUD micelles

Polymer	Average micellar size (nm)	Polydispersity (nm)	CMC (mg/l)	Zeta potential (mV)
PUD	50.3 ± 1.3	0.074 ± 0.006	0.2 ± 0.03	-42.98 ± 1.53

Data represent mean values ± standard deviation (SD).

### Drug release profile and kinetics

The capability of PUD nanomicelles to solubilize PTX in aqueous media was investigated using different ratios of drug to polymer. All the prepared solutions were clear opalescence, up to a certain ratio, and then precipitation was evident by visual inspection. The maximum solubilization of PTX occurred when the drug to polymer ratio was 1 (wt) to 1.25(%). At higher ratios, the solubility appeared to be lower. The maximum calculated loading content of PTX in PUD micelles was 80% (1 mg PTX per 1.25 mg polymer).

**Figure 4:**
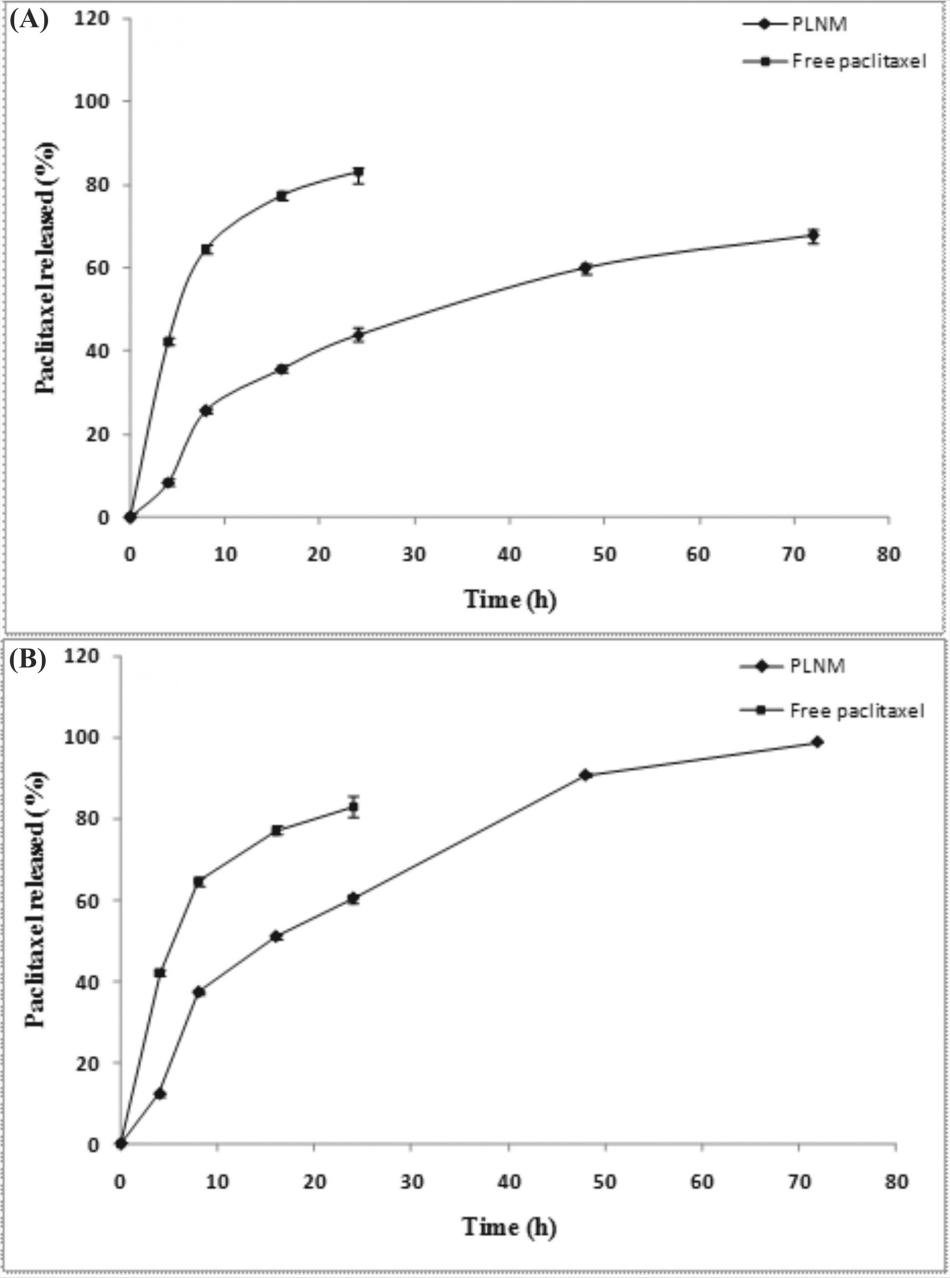
***In vitro *release profile of paclitaxel loaded nanomicelles**. **(A) **Drug release profile at pH 7.4. **(B) **Drug release profile at pH 5.4. All experiments were performed at 37°C. Data represent mean values ± SD (n = 3).

**Figure 5:**
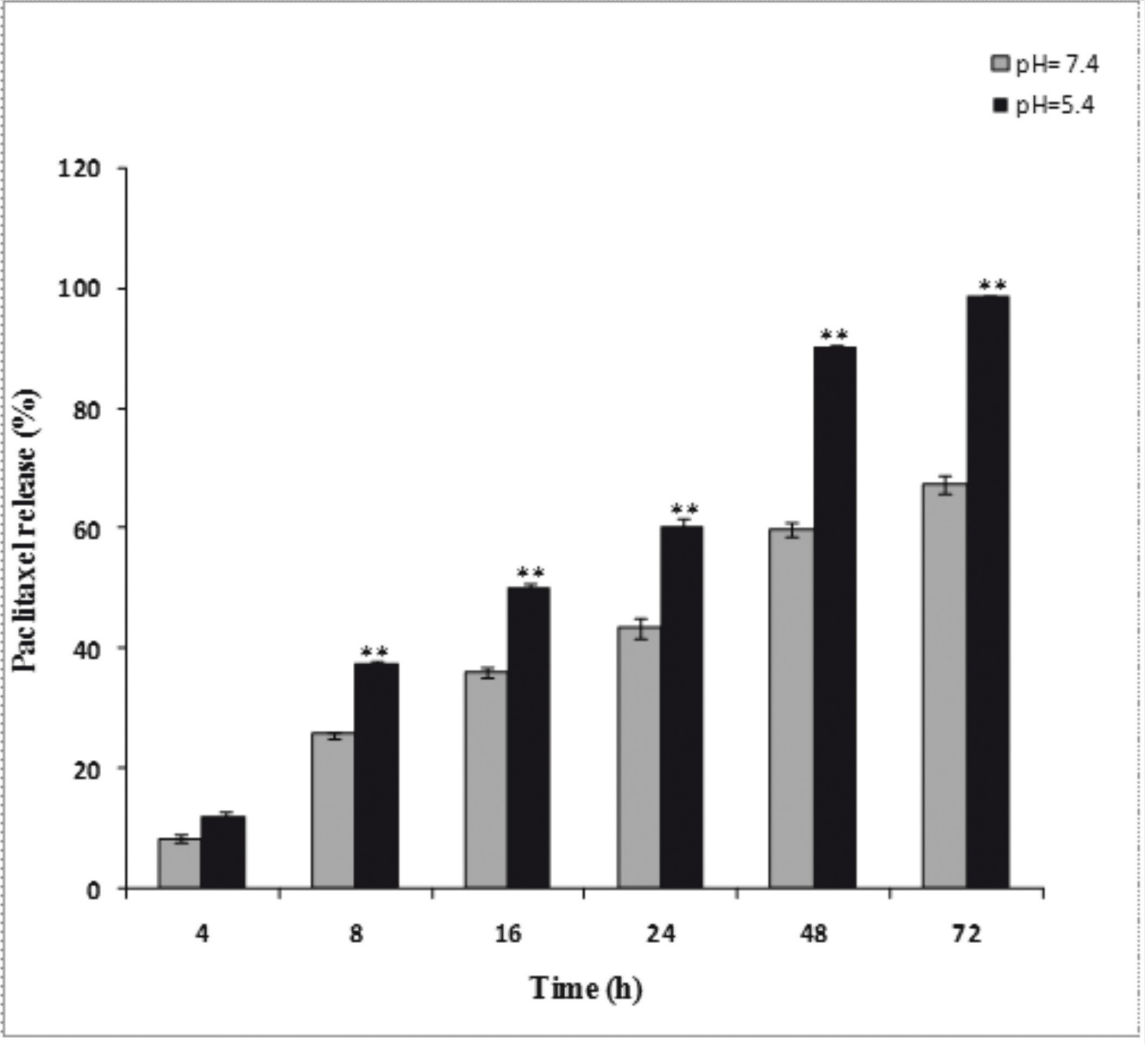
**Comparison of paclitaxel release profiles from nanomicelles at pH 7.4 and 5.4**. For comparison similarity factor (*f*_2_) was used. ** represents significant difference between two pH conditions (*f*_2 _< 50).

As shown in Figure 4, PTX release from polymeric micelles (over a 72 h period) was slower at pH 7.4 as compared to pH 5.4 (one-way ANOVA, P < 0.05). Comparison of the release profiles of free PTX revealed similar release profiles at pH 7.4 and 5.4 (*f_2 _*> 50). Under the studied conditions, 67.46% and 98.8% of physically loaded paclitaxel was released at pH 7.4 and 5.4, respectively (Figure 5). As shown in Table 2, the kinetic analysis of drug release data revealed that the release of PTX from nanomicelles was best fitted by Higuchi model (R^2 ^= 0.99), implying superiority of diffusion process [27].

**Table 2:** The release kinetics models of paclitaxel from nanomicelles at pH 7.4 and 5.4

Model name	Model	**R**^**2**^		reference
			
		pH 7.4	pH 5.4	
First order	ln (1-F) = - kft	0.974	0.780	[53]

Higuchi model	$\text{F}\;\text{ = }\;{\text{K}}_{\text{H}}\;\sqrt{\text{t}}$	0.990	0.984	[52,54]

Korsmeyer model	ln F = lnkp + plnt	0.948	0.936	[54]

Hixson-Crowell	$1\;-\;\sqrt[{3}]{1\;-\;\text{F}}\;\text{ = }\;{\text{K}}_{\text{1/2}}\;\text{t}$	0.985	0.899	[27]

Weibull	ln[-ln(1-F)] = - βln td + βlnt	0.974	0.780	[52,54]

Reciprocal powered time	$\frac{1}{F}\;-\;1\;=\;\frac{m}{t^{b}}$	0.947	0.973	[52]

More details on kinetic models for liberation of drugs from nanoparticles can be found in our previously published paper, see [52].

### MTT assay

**Figure 6:**
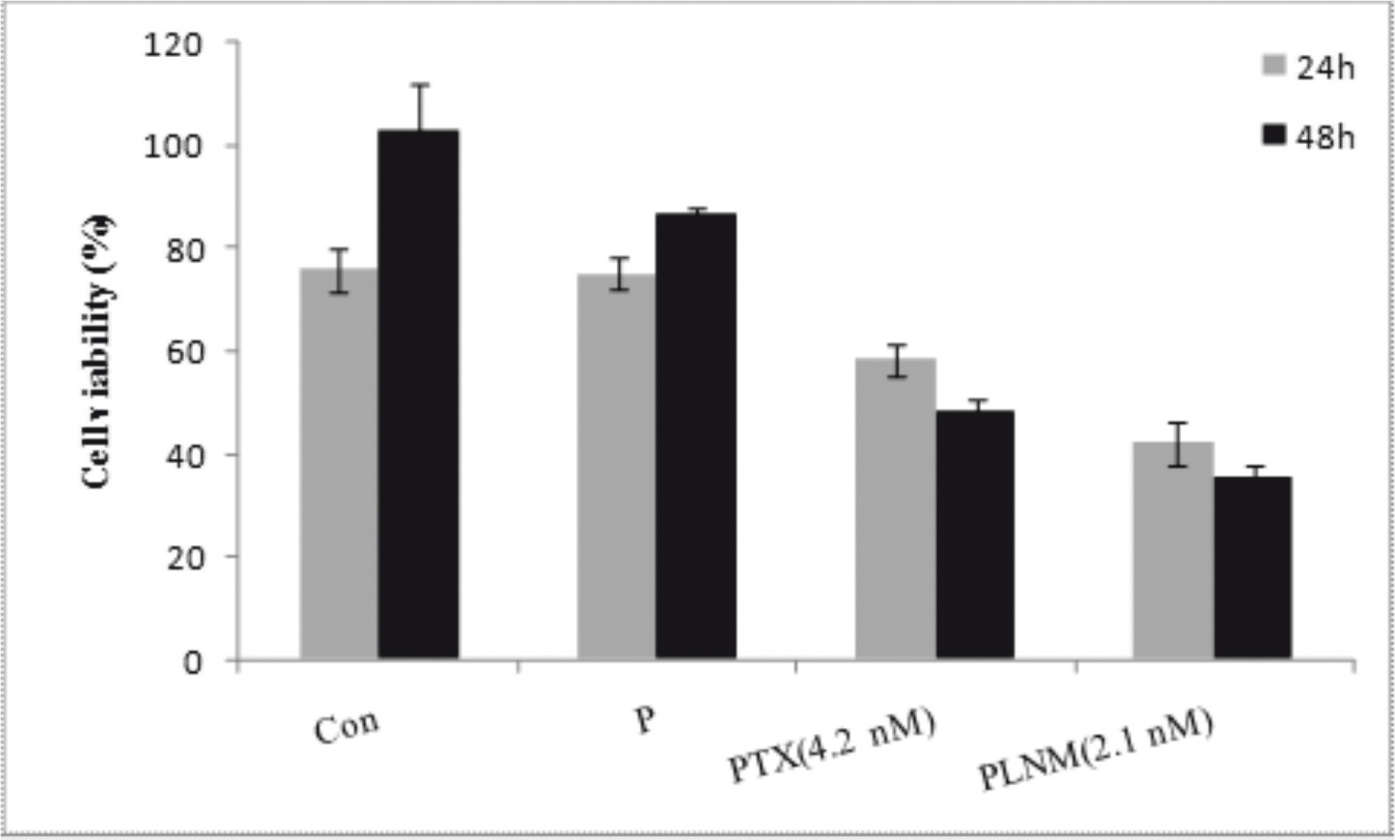
***In vitro *cytotoxicity of polymer, free paclitaxel and PTX loaded nanomicelles in MCF-7 cells**. Data represent cell viability of the treated cells after 24 and 48 h. Con: control, P: polymer, PTX: free paclitaxel (IC_50_, 4.2 nM), PLNM: paclitaxel loaded nanomicelles (included moiety of IC_50 _paclitaxel).

The MTT cytotoxicity assay showed cell viability changes in direct correlation with the concentration of both free paclitaxel and its polymeric formulations. As shown in Figure 6, in cultured MCF-7 cells, the PTX loaded nanomicelles resulted in higher cytotoxicity than free PTX (IC_50 _for MCF-7 cells is 4.2 nM) after 48 h incubation, while no noticeable cytotoxicity was observed for polymer itself at concentration used for the formulation of nanomicelles.

### Real time PCR assessment

**Figure 7:**
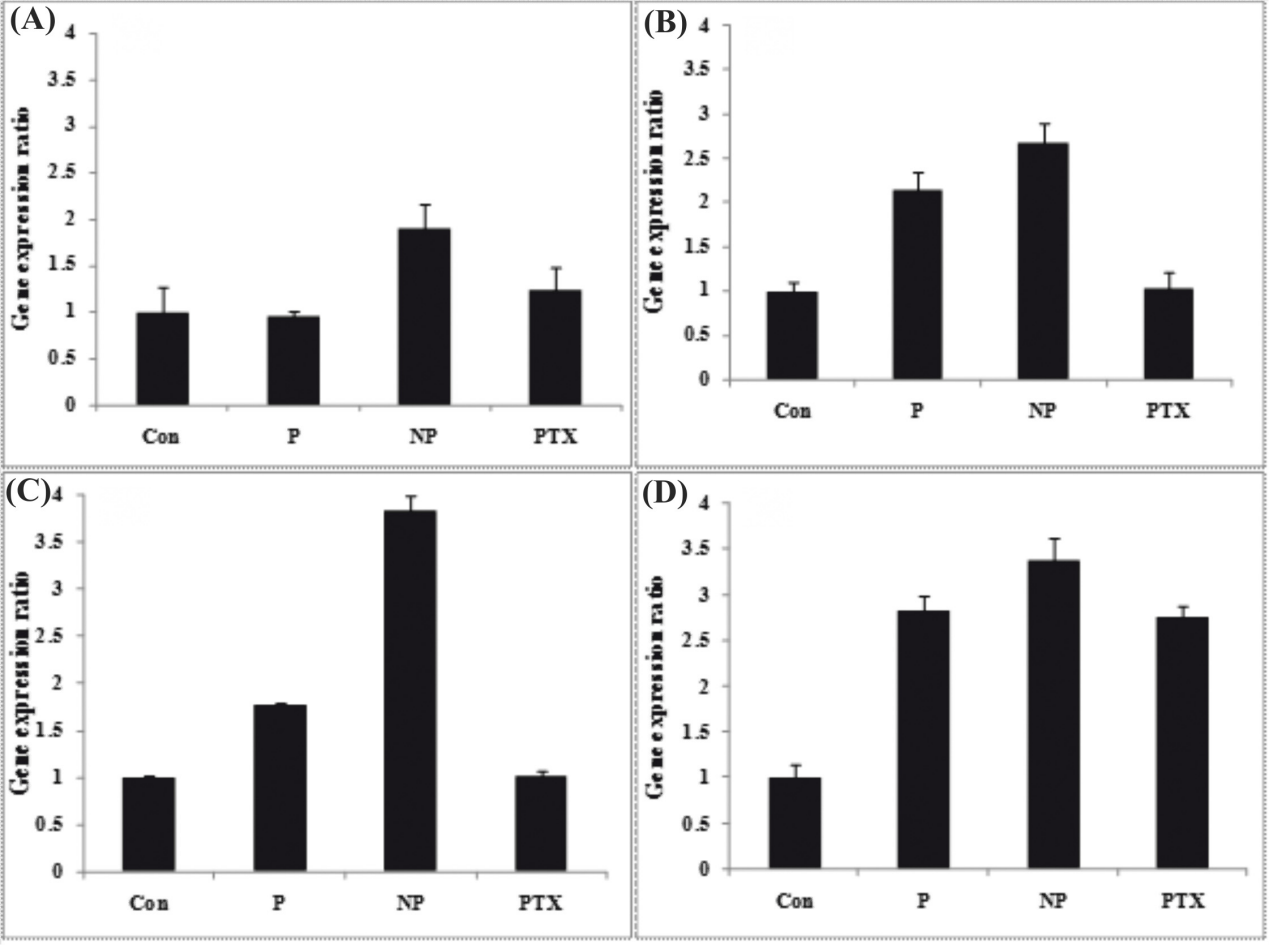
**Apoptosis related gene expression ratio in treated and untreated control cells**. **(A-D) **genes expression ratios of CYCS, CASP3, CAS9 and STAT1, respectively. Con: control, P: polymer, NP: paclitaxel loaded nanomicelles (included equal IC_50 _paclitaxel), PTX: free paclitaxel (IC_50_, 4.2 nM).

**Figure 8:**
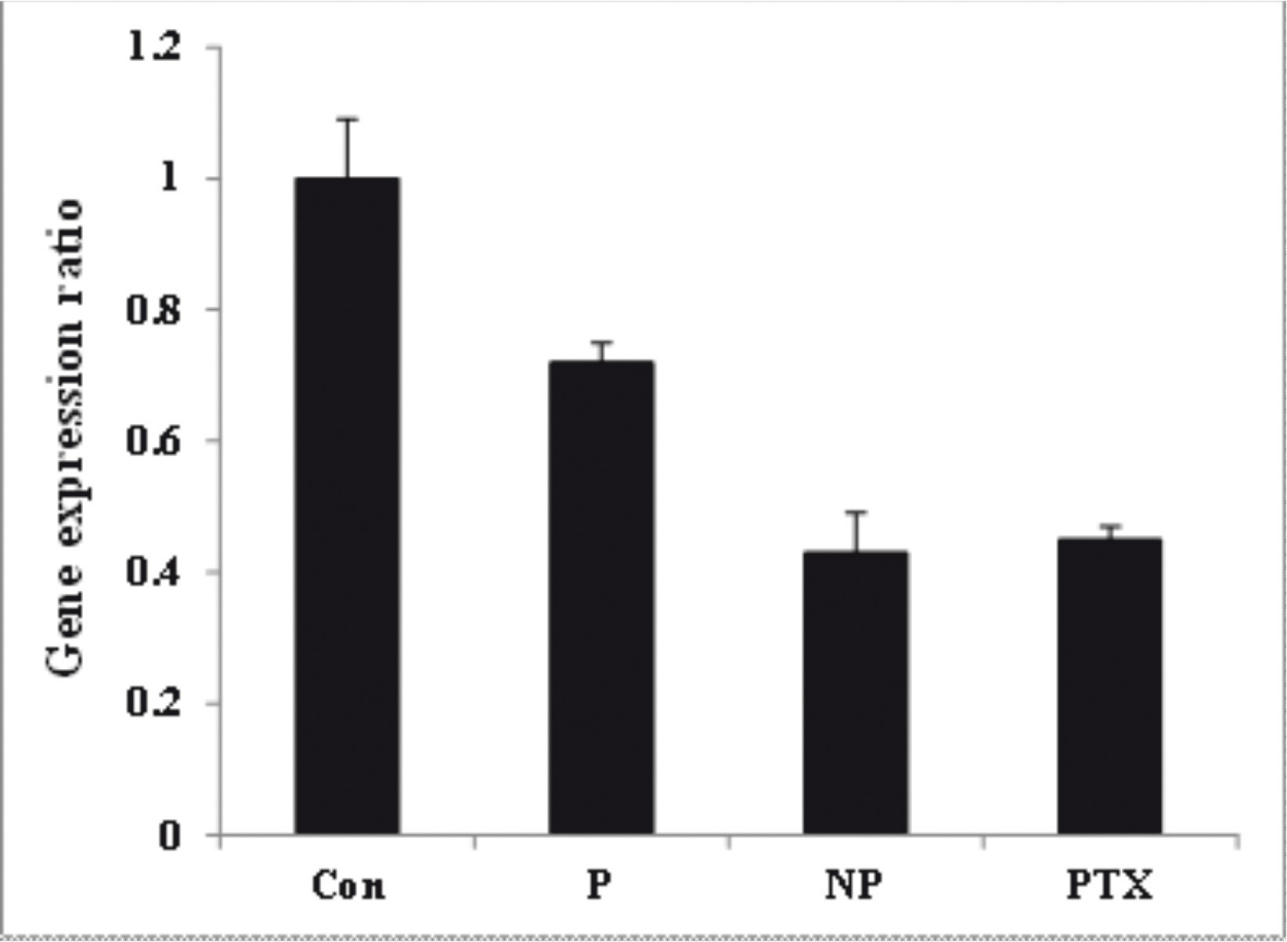
**Metastasis inducer gene (CTTN) expression ratio in treated and untreated control cells**. Con: control, P: polymer alone, NP: paclitaxel loaded nanomicelles (included equal IC_50 _paclitaxel), PTX: free paclitaxel (IC_50_, 4.2 nM).

As shown in Figure 7A, the expression of CYCS gene was not significantly altered by polymer or PTX, but significantly changed (upregulated) by the PTX loaded nanomicelles. The expression of CASP3 and CASP9 genes were altered (upregulated) in the cells treated with PU polymer itself and the PTX loaded nanomicelles after 48 h (Figures 7B and 7C). The expression of STAT1 gene was significantly upregulated in the cells treated with the PTX loaded nanomicelles, PTX and PU polymer (Figure 7D), while the expression of the metastasis inducer gene "CTTN" was significantly downregulated in all the treated cells as compared to the untreated control cells (Figure 8).

### Comet assay

**Figure 9:**
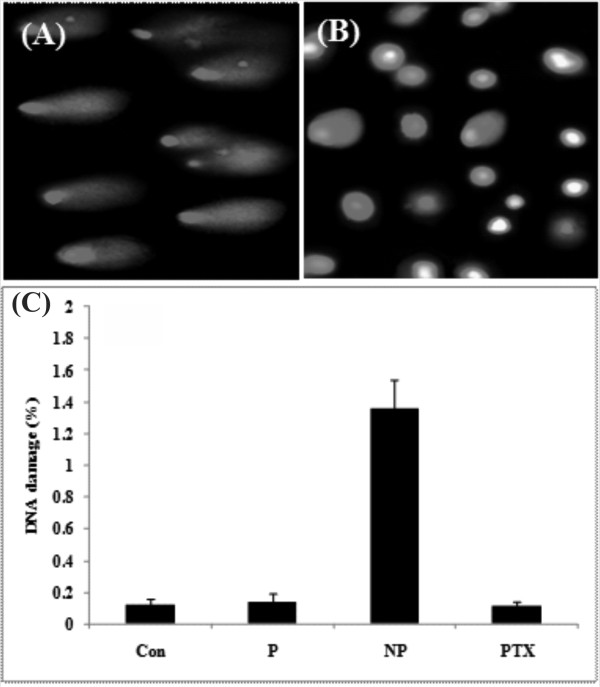
**DNA fragmentation by comet assay in treated and untreated control cells**. **(A) **Treated cells with PTX loaded nanomicelles. **(B) **Treated cells with PTX. **(C) **Normalized percentage of DNA fragmentation. Con: control, P: polymer, NP: paclitaxel loaded nanomicelles, PTX: free paclitaxel.

The comet assay was performed to detect possible DNA damage in the cells treated with the PTX loaded nanomicelles [28,29]. Figure 9 shows the comet assay for assessment of DNA integrity in the MCF-7 cells treated with the PTX loaded nanomicelles. The treated MCF-7 cells with polymer or PTX revealed no significant DNA fragmentation, but those cells treated with the PTX loaded nanomicelles showed DNA fragmentation.

## Discussion

The main objective of this research was to develop enzymatically bioactive polymeric nanomicelles for solubilization and efficient transportation of anticancer chemotherapies into cancer cells. To pursue such aim, we synthesized novel waterborne polyurethane with ability to form stable nanomicelles in aqueous media. The polymeric matrix was prepared based on commonly used method for preparation of waterborne polyurethane through introduction of anionic carboxylate ion in the backbone of polymers. For enhancing the stability of polymeric aqueous solution and controlling the molecular weight of polymer, the block ratio of OH and NCO containing moieties was adjusted to less than 1, and subsequently the free terminal NCO groups were blocked with phenol blocking agent. To the best of our knowledge, this is the first report on utilization of this polymeric nanocarrier for encapsulation and delivery of PTX. We speculate that the PTX loaded nanomicelles are less viable for uptake by RES. Thus, longer circulation of the PTX loaded nanomicelles in blood and greater EPR effects in tumor microenvironment are expected. And since the cosolvent mediated toxicity has been resolved, higher doses of PTX can be administered using these nanomicelles.

Having possessed suitable negative zeta potential charge, the PTX loaded PU based nanomicelles displayed no aggregation and lower level of CMC (Table 1). It has previously been reported that negatively charged nanoparticles can significantly internalize by the primary human alveolar immortal AT1 cells [30]. Further, hydrophilic nanomicelles were shown to stay in blood stream as long circulating drug delivery system [31,32], during which period the entrapped drug molecules can be protected from biological impacts (e.g., glomerular excretion, enzymatic degradation) and recognition of nanomicelles by RES. Also, the hydrophilic nanoparticles in range of 50-200 nm are deemed to be less prone to such biological impacts. The PTX loaded nanomicelles in our study displayed similar size range (~50 nm) (Table 1), and resulted in high physical stability without aggregation and/or sedimentation at room temperature for 9 days (Figure 3). The drug loading efficiency of the PTX loaded PU based nanomicelles was about 80%, demonstrating their higher drug encapsulation capacity. It should be stated that the high loading efficiency is generally considered as an advantage for the amphiphilic polymeric carriers because of reserving high amount of hydrophobic drug in hydrophobic core of micelle leading to an increased solubility of drug in aqueous media [33]. Our findings appeared to be somewhat similar to the results obtained from pluronic P105/L101 mixed polymeric micelles [34], however the smaller size of the PU based nanomicelles (i.e., 50 nm vs. 185 nm) may result in more efficient EPR effects. Ideally, a minimal leakage of the loaded drugs from drug nanocarriers should occur during circulation of the nanocarriers in blood stream. However, once taken up by cells, such nanocarriers should liberate the loaded drugs in the cytosol of the target cells to warrant the efficiency of chemotherapy. The release of drugs from pH-sensitive polymeric nanocarriers were shown to be triggered by the lower pH of the endosomal compartments [35]. Furthermore, biodistribution of pH-sensitive polymeric nanomicelles were shown to possess significantly longer blood circulation pattern and higher accumulation of drugs in solid tumors [35]. We looked at the drug release profiles of the PTX loaded nanomicelles at two different pH conditions (i.e., pH 7.4 and 5.4) and witnessed a faster PTX liberation profile at pH 5.5 (Figure 5). We assume that the ionic structure of PUD may fail at the lower pH value, perhaps as a result of transformation of carboxylate ionic groups to their acidic form and subsequent separation of polymeric carrier. In these nanomicelles, the release of PTX appeared to be dependent upon both diffusion and biodegradation processes. As shown in Figure 3, we monitored the particle sizes over the release time to confirm the possibility of degradation during the release period under an aqueous condition. The results showed no noticeable changes in the sizes of the nanomicelles measured by DLS at pH 7.4. This implies that drug release was mainly due to diffusion at pH 7.4, while diffusion and slight polymer degradation seemed to be the main mechanism of drug release at pH 5.4. Among the release kinetics models used for analysis of data (Table 2), the release of PTX from the nanomicelles was best fitted by Higuchi model (R^2 ^= 0.99) that describes the release of drugs from matrix as a square root of time dependent process based on Fickian diffusion law [27].

The cytotoxicity impacts of paclitaxel, polymeric carrier and drug loaded nanomicelles were also studied in the human breast cancer, MCF-7 cells. The PTX loaded nanomicelles showed a significant influence on the prevention of cell proliferation as compared to the untreated control and positive controls (i.e., PTX and polymers treated cells). Despite biologic impacts of different polymeric carriers (e.g., polyethylenimine) in target cells [36], the PU polymer alone showed no cytotoxic effects in the treated cells. The PTX alone induced somewhat cytotoxic effects in the treated cells; however such cytotoxicity was not comparable to that of the PTX loaded nanomicelles. Although we did not conduct a direct examination for the cellular uptake of nanomicelles, the cytotoxic effects and the gene expression changes induced by the PU nanomicelles can presumably indicate high uptake of the PTX loaded nanomicelles by the MCF-7 cells. Based upon physicochemical characteristics of these nanomicelles, we contemplate that these nanostructures can release the loaded drugs in the endosomal compartments in a pH-dependent manner. Similarly, pH-sensitive poly(2-tetrahydropyranyl methacrylate) [poly(THPMA)] nanospheres have recently been developed and shown higher cellular uptake potential with a pH-dependent release of the loaded drug (PTX) [37]. Micellar formulation of PTX using cholesterol-grafted poly(N-isopropylacrylamide-co-N, N-dimethylacrylamide-co-undecenoic acid) was reported to provide nanomicelles (~220 nm) with low CMC (~ 20 mg/L) and fast liberation of drug at pH 5.0 [38]. These PTX loaded nanomicelles were shown to induce toxicity against KB cells, in which a receptor-mediated endocytosis process was responsible for nanomicelles transportation [38]. Given the fact that enzymatic oxidation is the primary mechanism of biodegradation of the PU based nanomicelles [39], the PTX loaded nanomicelles used in our study may function as safer long circulating nanocarriers with ability of drug liberation into the cytosol.

To reveal the cytotoxic mechanism(s) of the PTX loaded nanomicelles, we looked at the gene expression profile of some pivotal genes related to apoptosis, in which the death signal is generated inside the cell after a chemical treatment leading to release of mitochondrial factors such as cytochrome c into the cytosol. The liberated cytochrome c interacts with APAF1 then triggers caspase 9. This complex is called apoptosome and it acts as a holoenzyme resulting in caspase 9 activation and finally leading to caspase 3 activation [40,41]. Thus, the release of cytochrome c into the cytosol of the target cells can basically be considered as a key regulatory step than can irreversibly coerce cells to commit an intrinsic apoptosis [42]. The STAT1 gene is represented by an anti-proliferative effect and consequent extrinsic apoptosis. Subsequent studies have shown that the STAT1 activates transcription of the CASP1 gene, a member of the protease family producing apoptosis and, in addition, activates transcription of the genes FAS and FASL, activators of the caspase system [43,44]. In our study, we examined the expression of some of these genes and witnessed significant overexpression of the genes studied (i.e., CYCS, CASP3, CASP9 and STAT1) in the treated cells with the PTX loaded nanomicelles even after 48 h. Interestingly, the expression of CYCS was not affected by the polymer itself as compared to the untreated control cells, which may be assumed as lack of intrinsic toxicity of polymer alone. However, STAT1 gene was somewhat overexpressed in the treated cells with the polymeric nanocarrier compared to the untreated control cells. We also observed overexpression of CASP3 and CASP9 induced by the polymer itself, which was not surprising since the overexpression of CASP3 and CASP9 genes may occur via CASP1 pathway activated by STAT1 gene (Figure 7). It should also be stated that a urethane compound diethyl-4,4'-methylenebis (N-phenylcarbamate) was shown to induce inhibitory effects on tubulin polymerization in the Chinese hamster cell lines (CHL and V79) and a human cancer cell line (HeLa S3), causing mitotic arrest even greater than that of PTX and colchicine and eliciting chromosome aberrations [45].

To examine possible DNA fragmentation, the comet assay was exploited with results showing significant DNA refraction in the treated cells with the PTX loaded nanomicelles (Figure 9). We speculate that the fragmentation of DNA occurs indirectly as a result of activation of apoptosis pathways and/or perhaps direct interaction of these nanomicelles with subcellular elements such as tubulin and DNA itself.

Metastasis in cancerous cells appears to be a crucial problem for cancer therapy. Thus, the impact of nanoformulations should be examined regarding their possible potential for induction of inadvertent intrinsic metastasis. To pursue this concept, we looked at the expression pattern of the CTTN gene (cortactin) which is an important gene for promoting lamellipodia and invadopodia formation as well as cell migration [46,47]. We found downregulation of CTTN gene in the treated cells with the PTX loaded nanomicelles (Figure 8), which may be considered as further indication for the safety of the PTX loaded nanomicelles from this point of view.

## Conclusion

To develop bioactive biocompatible nanomicelles for delivery of anticancer agents such as paclitaxel, an oxidatively biodegradable polyurethane polymer was exploited to synthesize waterborne nanomicelles containing PTX. The PU nanomicelles showed considerably appropriate physicochemical (e.g., ~50 nm in diameter, 0.2 mg/L CMC, suitable negative zeta potential charge, high stability, very low aggregation, high loading efficiency, and pH-sensitivity for PTX release) and biological (e.g., gene expression alterations) properties. The PTX loaded nanomicelles were significantly taken up by the MCF-7 cells resulting in significant activation of important genes related to the cell death program such as cytochrome c. Based upon these findings, the self-assembled waterborne polyurethane nanomicelles may be considered as an appropriate drug delivery nanocarrier candidate for further translational investigations.

## Methods

### Materials

Poly(tetramethylene ether) glycol (PTMEG, OH functionality = 2.0, Mn: 2000 g mol^-1^), imethylol propionic acid (DMPA), RPMI 1640 complete growth media, 3-(4,5-dimethylthiazol-2-yl)-2,5-diphenyltetrazolium bromide (MTT), normal melting point agarose, low melting point agarose, fetal bovine serum, paclitaxel, penicillin, streptomycin and TRI Reagent^® ^were obtained from Sigma-Aldrich Chemical Co. (Poole, UK). Toluene diisocyanate (TDI mixture of 2,4 and 2,6-isomers), phenol, triethylamine (TEA) and N-Methyl-2-pyrrolidon (NMP), dimethyl sulfoxide (DMSO), acetonitrile, pyrene, sodium salicylate, glycine, NaCl, chloroform, diethylpyrocarbonate (DEPC), Trizma base, Triton-X100, ethylenediaminetetraacetic acid (EDTA) citrate buffer salts and phosphate buffer salts were obtained from Merck (Darmstadt, Germany). Moloney murine leukemia virus (MMLV) reverse transcriptase, MMLV buffer with dithiothreitol (DTT) and random hexamer primer were purchased from Fermentas Life Science (Burlington, Canada) and Brilliant^®^SYBR^® ^Green I PCR master mix was prepared from Stratagene (La Jolla, CA, USA).

### Synthesis of polyurethane dispersion (PUD)

PTMEG (0.06 mol, dried at 80°C under vacuum for 24 h) and DMPA (0.06 mol) in equal weight of NMP were placed into a four-necked reaction kettle equipped with a mechanical stirrer, heating mantle, reflux condenser, dropping funnel and N_2 _inlet and outlet. The reaction mixture was stirred at 75°C under N_2 _atmosphere. After complete mixing, vacuum distillation purified TDI (0.16 mol) was dropped into the reactor at such a rate that the reaction temperature would not surpass 75°C. The temperature was then increased to 85°C and the reaction was continued till the NCO content reached the theoretical value as determined by dibutyl amine back titration method. Then the reaction mixture was cooled down to 60°C and followed by the slow addition of phenol (0.08 mol) diluted with acetone (150 ml). The reactions were carried out until NCO peak disappeared in the FTIR spectrum. The blocked polyurethane pre-polymers were cooled to 50°C and the neutralizing agent, TEA (0.06 mol) was added and allowed to react to form ionomer for 30 min. Finally, DD water was added to accomplish the dispersion under vigorous stirring. A uniform PUD was obtained, from which the acetone was removed under low vacuum at 60°C. The final solution was diluted with water in a way that each ml of solution contains 125 mg PU. The scheme of these reactions is illustrated in Figure 1.

### Characterization of PUD

PUDs were characterized by spectroscopic methods. Fourier transform infrared (FTIR) spectra were obtained using Bruker IFS 48 instrument (Bruker Optik GmbH, Germany). All spectra were taken under air as a function of time with 16 scans at a resolution of 4 cm^-1 ^and a spectral range of 4000-5000 cm^-1^. Nuclear magnetic resonance (^1^HNMR) spectra were recorded on a Bruker model AVANCE DPX 400 MHz instrument (Bruker Optik GmbH, Germany) with CDCl_3 _as solvent. Spectra were averaged from 8 transients and calibrated by proton lock method.

### Physical encapsulation of paclitaxel into PUD

Sonication method was used to prepare waterborne polyurethane nanomicelles with highly loaded PTX. For this purpose, PTX (0.1-1.25 mg) was dissolved in DMSO (25 μl) and added to the fixed concentration of polymer in the deionized water (1.25 mg per each ml of solution). The mixture was sonicated (at 250 kJ) for 10 sec. This colloidal solution was then centrifuged at 21000 × *g *(SIGMA Laborzentrifugen GmbH, Germany) for 10 min to remove free, unloaded PTX molecules. The supernatant solution was analyzed for paclitaxel content using Pharmacia Biotech HPLC system (San Francisco, CA, USA) equipped with C18 Column (4.6 × 250 mm, 5 μm). The mobile phase was acetonitrile/H2O (60:40) with flow rate of 1.0 ml/min and injection volume of 20 μl. Detection of paclitaxel was accomplished at 227 nm. The PTX loading rate (w/w %) and the encapsulation efficiency (w/w %) were calculated as previously described [48].

### Determination of critical micelle concentration (CMC)

A change in the fluorescence excitation/emission spectra of pyrene in the presence of designated concentration of the PUD was used to measure the CMC according to a method described previously [49]. Briefly, pyrene was dissolved in DMSO to provide a final concentration of 6 × 10^-7 ^M. Aqueous polymeric solutions with concentration ranging from 0.1-1.5 mg/L were then added and samples were sonicated for 10 sec, just before the fluorescence emission measurements. The excitation spectrum of pyrene for each sample was obtained at room temperature using Perkin Elmer LS55 fluorescence spectrophotometer (Perkin Elmer, Rockville, USA). The emission wavelength and the excitation/emission slit were set at 390 nm and 5 nm, respectively. The quenching intensity of the emission peak at 394 nm was plotted against various polymer concentrations. The CMC was measured from a decrement emission intensity peak (394 nm) at the onset of micellization.

### Determination of size and zeta potential of micelles

The average diameter, size distribution and zeta potential of the prepared nanocarriers were estimated by dynamic light scattering (DLS) using Brookhaven Zeta PALS (Brookhaven Instruments Corporation, Austin, USA) after centrifugation at 21000 × *g*, pH 7.4 for 10 min (SIGMA Laborzentrifugen GmbH, Germany).

### Evaluation of the physical stability of nanocarrier

PTX loaded PUD micellar solution (1 mg/ml) was prepared in phosphate buffer (0.01 M, pH 7.4) and left for 9 days at room temperature. At different time points, the hydrodynamic diameter and the polydispersity of the micellar solution was assessed using Brookhaven Zeta PALS (Brookhaven Instruments Corporation, Austin, USA).

### Determination of paclitaxel release profile from polymeric micelles

The amounts of PTX released from loaded micelles were determined using phosphate buffer (0.01 M, pH 7.4) and citrate buffer (0.01 M, pH 5.4) containing 2 M sodium salicylate at 37°C [50,51]. The experiments were initiated by adding free or micellar PTX solution to the buffer to provide a final concentration of 0.5 mg/ml of PTX. Then, 5 ml of the micellar solution was transferred into a dialysis bag (Spectrapor, MW cutoff 3500 gmol^-1^) and placed into 500 ml of phosphate buffer (0.01 M, pH 7.4) and citrate buffer (0.01 M, pH 5.4). The release study was performed at 37°C using a shaking water bath (GFL, Burgwedel, Germany). At the designated time intervals, considering sink condition, sampling was performed. In each time point, 30 μl of sample was withdrawn, freeze dried and dissolved in 60 μl of methanol. Then, 20 μl aliquot was injected into HPLC to determine the quantity of released PTX. The accumulated release was calculated using following equation:

$$R\;=\;\frac{V\sum_{i}^{n-i}C_{i}\;+\;V_{o}\;C_{n}}{m_{drug}}$$

Where, R is the accumulated release (%), V is the sampling volume, V_0 _is the initial volume, C_i _and C_n _are the paclitaxel concentrations, i and n are the sampling times, and m_drug _is the mass of paclitaxel in PUD nanomicelles.

The release profiles were compared using similarity factor *f*_2_. The profiles were significantly different when *f_2 _*was less than 50 as described previously [28]. Various important kinetic models (Table 2) were exploited to address the release pattern of the PTX from nanomicelles as we described previously [52].

### In vitro toxicity of paclitaxel loaded PUD micelles

MTT assay was recruited for cytotoxicity assessment of PTX loaded PUD micelles in human breast cancer, MCF-7 cells. Briefly, cells were cultured at a seeding density of 4.0 × 10^4^/cm^2 ^in 96-well microplate. The cultivated cells were maintained in a humidified atmosphere (5% CO2/95% air) at 37°C with medium comprising of RPMI 1640 complete growth media supplemented with 10% FBS, 100 units/ml penicillin and 100 μg/ml streptomycin. At 50% confluence (24 h post seeding), the cultured cells were treated with several concentrations of PTX loaded (2.1 nM) and unloaded nanomicelles as well as free PTX (IC50, 4.2 nM) over different incubation periods (24 and 48 h). Then the medium was replaced with 200 μl fresh media containing 50 μl of MTT solution (5 mg/ml in PBS) and the cells were incubated for an additional 4 h at 37°C. After incubation period, the media/MTT mixture was carefully removed and 200 μl of DMSO plus 25 μl of Sorenson's glycine buffer (0.1 M glycine, 0.1 M NaCl, pH 10.5) were added to each well. The absorbance of each well was measured after 10 second shaking, employing a microplate reader ELX808 (Bioteck, Vermont, VT, USA) at 570 nm. The mean and standard deviation values for each treatment were determined and converted to the percentage of viable cells relative to the control.

### Quantitative real time PCR

#### Total RNA extraction

For RNA analysis, MCF-7 cells were lysed using TRI Reagent^® ^according to manufacture guidelines. In brief, 48 h post treatment or untreated control monolayer cells were lysed by adding desired amount of TRI Reagent^® ^(2 ml per 25 cm^2 ^T-flask). The lysates were homogenized and transferred to RNAse/DNAse-free microtubes. Chloroform (0.2 ml per each ml of TRI Reagent™ used for lysing) was added to each sample, and the mixture was vortexed. After maintaining at room temperature for 5 min, the samples were centrifuged at 12000 × *g*, 4°C and 10 min and the colorless upper aqueous phase was carefully separated and mixed with ice-cold iso-propanolol (0.5 ml per each ml of TRI Reagent^® ^used initially). The mixture was centrifuged at 12000 × *g*, 4°C for 10 min, yielding total RNA pellet that was washed with 75% ethanol (×3). The air dried samples were dissolved in DEPC treated water and tested qualitatively and quantitatively prior to its use for RT-PCR experiments.

#### RT reaction and cDNA preparation

The isolated RNA was reverse transcribed to cDNA using MMLV reverse transcriptase. For RT reaction, 1 μl RNA (1 μg/μl) was mixed with master mix [DEPC treated water 13 μl, dNTP's (10 μM) 2 μl, MMLV buffer with DTT 2 μl, random hexamer primer (pdN6; 400 ng/μl) 0.5 μl], and denatured at 95°C for 5 min. The sample was then cooled down to 4°C for 5 min using ice-bath. Then 1 μl MMLV (200 U/μl) and 0.5 μl RNasin (40 U/μl) were added to the sample and the mixture was incubated using following thermocycling program: 10 min at 25°C, 42 min at 42°C, and 5 min at 95°C. The prepared cDNA templates were used for real time PCR experiments.

#### Real time PCR

Primers were designed from published Gene Bank sequences using Beacon Designer 5.01 (Premier Biosoft International, http://www.premierbiosoft.com) and listed in Table 3. All amplification reactions were performed in a total volume of 25 μl using iQ5 Optical System (Bio-Rad Laboratories Inc., Hercules, USA). Each well contained: 1 μl cDNA, 1 μl primer (100 nM each primer), 12.5 μl 2× Power SYBR Green PCR Master Mix (Applied Biosystems, Foster City, USA), and 10.5 μl RNAse/DNAse free water. Thermal cycling conditions were as follow: 1 cycle at 94°C for 10 min, 40 cycles at 95°C for 15 sec, 56-62°C (annealing temperature, see Table 3 for details) for 30 sec, and 72°C for 25 sec. Interpretation of the result was performed using the Pfaffle method and the threshold cycle (Ct) values were normalized to the expression rate of GAPDH as a housekeeping gene. All reactions were performed in triplicate and negative controls were included in each experiment.

**Table 3:** Real time PCR genes and their forward/reverse primers

Gene name & Accession No	Primer sequence	T_m _(°C)	T_a _(°C)	Size (bp)
GAPDH (NM_002046.3)	F:5'-AAGCTCATTTCCTGGTATGACAACG-3' R:5'-TCTTCCTCTTGTGCTCTTGCTGG-3'	61.3 62.4	59	126
CASP3 (NM_004346.3)	F: 5'-TGCCTGTAACTTGAGAGTAGATGG-3' R: 5'-CTTCACTTTCTTACTTGGCGATGG-3'	61 61	56	172
CYCS (NM_018947.4)	F: 5'- ACCTTCCATCTTGGCTAGTTGTG-3' R: 5'- ATCGCTTGAGCCTGGGAAATAG-3'	59.3 59	58	129
CASP9 (NM_001229.2)	F: 5'- TGCTGCGTGGTGGTCATTCTC-3' R: 5'- CCGACACAGGGCATCCATCTG-3'	61.8 63.7	62	94
STAT1 (NM_007315.3)	F: 5'- TCATCAGCAAGGAGCGAGAG-3' R: 5'- TCAGGGAAAGTAACAGCAGAAAG-3'	59.4 58.9	56	196
CTTN (NM_005231.3)	F: 5'- GCCGACCGAGTAGACAAGAGC-3' R: 5'- ATTTGCCGCCGAAACCTTTGG-3'	63.7 59.8	59	100

T_m_: Melting temperature; T_a_: Annealing temperature

#### Comet assay

To detect possible DNA damage, the comet assay was carried out. Prior to the comet assay, the trypan blue based viability test was performed to ensure about the viability of cells (> 70%) [28,29]. Briefly, the slides were precoated with normal melting point agarose (1% in PBS; 0.1 M and pH 7.2) and dried at room temperature. Cells (5.0 × 10^5^/mL) were mixed with low melting point agarose (0.5% in PBS; 0.1 M, pH 7.2 and 37°C) and one drop of mixture was placed on precoated slide and covered with lamella. The slides were kept in a freezer (-20°C) for 10 min and then placed in lysis buffer (NaCl 2.5 M, EDTA 100 mM, Trizma base 10 mM, Triton X-100 1%, DMSO 10%, pH 10, and 4°C) for 4 h. The slides were washed with distilled water (×3) and placed in contractive buffer (Tris base 0.4 M and pH 7.5) for 10 min, and then in electrophoresis buffer (30 ml NaOH 10 N and 5 ml EDTA 200 mM in 1 L distilled water; pH 13) for 20 min. The slides were electrophoresed at 25 A and 300 mV condition for 40 min using electrophoresis buffer, then placed in staining buffer (ethidium bromide 0.02 mg/ml) for 5 min and washed (×3) with distilled water. The fluorescent images were obtained using Olympus IX81 microscopes (Olympus Optical, Tokyo, Japan) and DNA damage was quantified using Casp software (CaspLab, University of Wroclaw, Institute of Theoretical Physics).

#### Statistical analysis

One way ANOVA with an appropriate multiple-comparison test or Student's t-test were employed for statistical analysis using SPSS and MSTATC. A *p*-value less than 0.05 was considered statistically significant. Data were represented as mean ± standard deviation (SD) of triple measurements.

## Abbreviations

CMC: critical micelle concentration; DEPC: diethylpyrocarbonate; DMPA: imethylol propionic acid; DMSO: dimethyl sulfoxide; DTT: dithiothreitol; EDTA: ethylenediaminetetraacetic acid; EPR: enhanced permeation and retention; MMLV: Moloney murine leukemia virus; MTT: 3-(4,5-dimethylthiazol-2-yl)-2,5-diphenyltetrazolium bromide; NMP: N-Methyl-2-pyrrolidon; PCR: polymerase chain reaction; PTMEG: poly (tetramethylene ether) glycol; PTX: paclitaxel; PUD: polyurethane dispersion; PU: polyurethane; RES: reticuloendothelial system; TDI: toluene diisocyanate; TEA: triethylamine; THPMA: tetrahydropyranyl methacrylate.

## Competing interests

The authors declare that they have no competing interests.

## Authors' contributions

HN, AYK and YO designed the study. AYK and HY contributed the synthesis and characterization of polymers. AYK and JB carried out the cellular/molecular based experiments. HN and AYK performed all statistical analysis. AYK and YO performed kinetics modeling. AYK and YO drafted the manuscript. HN, AYK, JB and YO revised the manuscript. HN and YO acted as correspondence. All authors have read and approved the final manuscript.
